# Determination of Antidepressants in Human Plasma by Modified Cloud-Point Extraction Coupled with Mass Spectrometry

**DOI:** 10.3390/ph13120458

**Published:** 2020-12-12

**Authors:** Elżbieta Gniazdowska, Natalia Korytowska, Grzegorz Kłudka, Joanna Giebułtowicz

**Affiliations:** 1 Łukasiewicz Research Network, Industrial Chemistry Institute, 8 Rydygiera, 01-793 Warsaw, Poland; elzbieta.gniazdowska@wum.edu.pl; 2 Department of Bioanalysis and Drugs Analysis, Doctoral School, Medical University of Warsaw, 61 Żwirki i Wigury, 02-091 Warsaw, Poland; 3 Department of Bioanalysis and Drugs Analysis, Faculty of Pharmacy, Medical University of Warsaw, 1 Banacha, 02-097 Warsaw, Poland; nkorytowska@wum.edu.pl (N.K.); grzegorz.kludka@gmail.com (G.K.)

**Keywords:** sample preparation, LC–MS/MS, antidepressant, bioanalytical methods

## Abstract

Cloud-point extraction (CPE) is rarely combined with liquid chromatography coupled to mass spectrometry (LC–MS) in drug determination due to the matrix effect (ME). However, we have recently shown that ME is not a limiting factor in CPE. Low extraction efficiency may be improved by salt addition, but none of the salts used in CPE are suitable for LC–MS. It is the first time that the influences of a volatile salt—ammonium acetate (AA)—on the CPE extraction efficiency and ME have been studied. Our modification of CPE included also the use of ethanol instead of acetonitrile to reduce the sample viscosity and make the method more environmentally friendly. We developed and validated CPE–LC–MS for the simultaneous determination of 21 antidepressants in plasma that can be useful for clinical and forensic toxicology. The selected parameters included Triton X-114 concentration (1.5 and 6%, *w/v*), concentration of AA (0, 10, 20 and 30%, *w/v*), and pH (3.5, 6.8 and 10.2). The addition of 10% of AA increased recovery twice. For 20 and 30% (*w/v*) of AA, three phases were formed that prolonged the extraction process. The developed CPE method (6% Triton X-114, 10% AA, pH 10.2) was successfully validated through LC–MS/MS simultaneous determination of 21 antidepressants in human plasma. The linearity was in the range of 10–750 ng/mL (r^2^ > 0.990).

## 1. Introduction

Cloud-point extraction (CPE) is a modification of liquid–liquid extraction (LLE) that is more friendly to the environment and users, mainly due to lower consumption of organic solvents according to the global trend of "green chemistry.” CPE has also advantages over solid phase extraction (SPE), such as faster and cheaper optimization processes and no need for expensive equipment [1]. In CPE, the surfactant (such as Triton X-114) in a concentration above the critical micelle concentration (CMC) can exist as a homogeneous isotropic liquid, which separates into two isotropic phases with different concentrations of surfactant. Sample ingredients are separated into the surfactant micelle-rich phase (hydrophobic components when nonionic surfactant is used) and the micelle-poor phase (hydrophilic components) [2].

Nowadays, the most reliable technique of pharmaceutical determination is liquid chromatography coupled to mass spectrometry (LC–MS). However, there are only a very few papers reporting LC–MS coupled with CPE in drug determination, i.e., in the determination of memantine in rat plasma [3], and bisoprolol [4], antazoline [1], abacavir, efavirenz, lamivudine, and belfinavir in human plasma [5]. The disadvantage of the CPE–LC–MS, contrary to LLE–LC–MS, is the lower extraction efficiency (recovery). Recovery for bisoprolol was 46−61% using CPE and 74−86% using LLE [4]. In CPE methods coupled with other techniques than mass spectrometry, non-volatile salts such as NaCl [6,7,8,9], Na_2_SO_4_ [10], Na_2_B_4_O_7_ [11], and (NH_4_)_2_SO_4_ [12] are used to improve the efficiency of extraction. However, these salts are incompatible with LC–MS due to possible matrix effect (ME) and deposition in the ion source. Moreover, they crystallize in the capillary lumen resulting in the additional need for cleaning and maintenance of LC–MS. The alternative approach is the usage of volatile salts such as ammonium acetate (AA) and formate. These salts are the commonly used modifiers of a mobile phase in positive ionization mode in LC–MS. However, there are no reports on their application in CPE–LC–MS. 

Determination of antidepressants in biological samples is necessary for the effective and safe treatment of depression, a common mental disorder. According to World Health Organization (WHO), more than 264 million people of all ages suffer from depression globally. This disease is one of the most serious public health problems [13]. Moreover, the treatment of depression is a complex issue, sometimes being associated with dysphoric, mixed, agitated, or psychotic states that can increase suicidal risk [14]. Moreover, antidepressants are frequently used together with other legal or illegal drugs and can result in a synergy of symptoms and intoxication. The drugs can be also used in intentional poisoning [15,16]. The antemortem screening analysis can be useful in cases of suspected poisoning or vehicle accidents in clinical or forensic toxicology investigations. The screening method should allow one to rapidly determine and quantify the wide spectrum of compounds. Thus, the method of simultaneous determination of antidepressants from different classes is needed. 

There are many LC methods for the determination of antidepressants using various detectors, e.g., UV–Vis, photodiode-array (PDA), diode-array (DAD), and fluorescence detector. Recently, MS has been also extensively employed in the analysis of complex biological samples especially [17]. Since detectors based on UV–visible spectrometry are universal, less expensive, and less complicated than LC–MS, they are commonly used in routine laboratories. They frequently provide a satisfactory limit of quantitation for antidepressants in plasma (even 5 ng/mL for some compounds) [18], due to aromatic rings (e.g., phenyl group and naphthalene group) and other major UV chromophores present in their structures. Those detectors are also applied in multi-methods, permitting one to simultaneously determine up to 11 antidepressants in plasma (LC–PDA) [19]. However, the conventional detectors are less selective than LC–MS, which is crucial in forensic and clinical toxicology when in samples many legal and illegal compounds may occur. Thus, LC–MS remains the gold standard, especially in legal medicine. Since the method has a drawback of matrix effects and ion suppression issues, the careful validation needs to be performed [17].

LC–MS methods of antidepressant determination use electrospray ionization in positive ion mode (ESI+) [20,21,22,23,24,25,26,27,28], atmospheric pressure chemical ionization in positive ion mode (APCI+) [29], or QTOF MS in positive ionization mode with a DuoSpray ion source [30]. Samples were prepared using LLE [20,26,29], microextraction by packed sorbent (MEPS) [28], protein precipitation (PP) [25,30], SPE [22], on-line SPE [21,23], capillary restricted-access media (RAM) [27], and online-restricted access molecularly imprinted solid-phase extraction (RAMIP-BSA) [24]. All methods require the use of environmentally harmful organic solvents. As an alternative, we aim to develop the environmentally friendly CPE that can be coupled with mass spectrometry. The novel CPE protocol provides better recoveries than the previous studies reporting the use of CPE–LC–MS [1,4].

This study aimed to examine the effects of one volatile salt AA’s addition on recovery and the matrix effect of the CPE–LC–MS method. To make the extraction method more environmentally friendly, we used ethanol instead of methanol or acetonitrile to reduce the sample viscosity at the last point of sample preparation. We have developed the method for the simultaneous determination of 21 major antidepressants. The compounds were from four different classes—i.e., non-selective monoamine reuptake inhibitors (N06AA): amitriptyline, clomipramine, desipramine, doxepin, imipramine, maprotiline, nortriptyline, opipramol, protriptyline, and trimipramine; selective serotonin reuptake inhibitors (SSRIs)(N06AB): citalopram, fluoxetine, fluvoxamine, paroxetine, and sertraline; monoamine oxidase A inhibitors (N06AG): moclobemide; and other antidepressants (N06AX): mianserin, mirtazapine, tianeptine, trazodone, and venlafaxine. The selected pharmaceuticals have different structures and chemical properties, and logP and pKa range from 1.5 to 5.2 and 6.0 to 10.5, respectively. The analyzed parameters for the simultaneous determination of the compounds were the concentration of non-ionic surfactant Triton X-114, sample pH, and AA concentration. Finally, validation parameters were compared with several reported methods.

## 2. Results and Discussion

### 2.1. Development of Cloud-Point Extraction 

**Figure 1:**
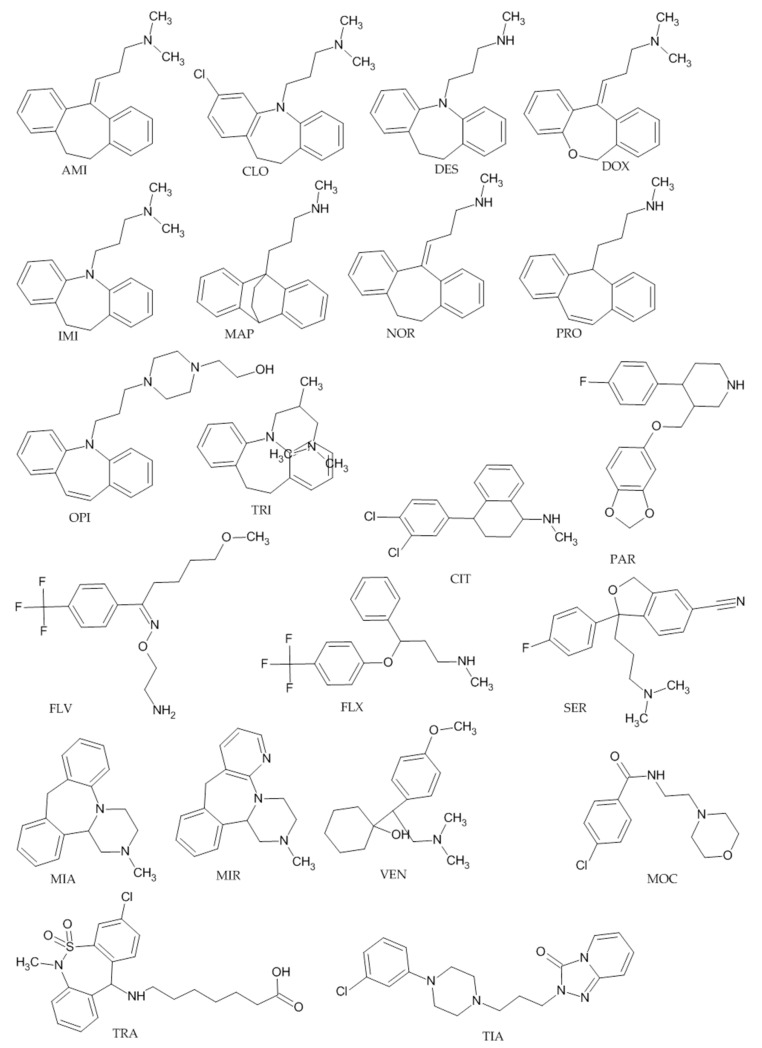
Chemical structures of antidepressants studied in the experiment.

The analytical method was developed for determination of such antidepressants as amitriptyline (AMI), citalopram (CIT), clomipramine (CLO), desipramine (DES), doxepin (DOX), fluoxetine (FLX), fluvoxamine (FLV), imipramine (IMI), maprotiline (MAP), mianserin (MIA), mirtazapine (MIR), moclobemide (MOC), nortriptyline (NOR), opipramol (OPI), paroxetine (PAR), protriptyline (PRO), sertraline (SER), tianeptine (TIA), trazodone (TRA), trimipramine (TRI), and venlafaxine (VEN) in human plasma by LC–MS/MS (Figure 1).

The initial factors of extraction included pH 6.8, temperature 60°C, and 20% of AA. To reach pH 6.8, only water was used. Thus, pH 6.8 was selected to reduce the time of the process, reduce the cost, and diminish the risk of contamination. AA concentration was selected as a medium value from all tested in the experiment. The level of antidepressants corresponded to a plasma concentration of 100 ng/mL.

Many factors affect CPE efficiency. In this paper, the influences of surfactant concentration (Triton X-114), volatile salt (AA—0, 10, 20, and 30% (*w/v*)), and extraction pH (about 3.5, 6.8, 10.2) was tested to select the optimal conditions for the simultaneous determination of 21 antidepressants. Other parameters, i.e., volume of reagents and equilibrium time, were used as described previously [2,31]. However, in the current study higher temperature was selected (60 °C). The optimal temperature for CPE is 15–20 °C greater than the cloud point of the surfactant, which in the case of Triton X-114 is 23 °C, and was reviewed to be 40–60 °C [32]. With an increase in temperature, the efficacy of the extraction increases, and the volume of the surfactant-rich phase decreases due to the disruption of the hydrogen bonds and the dehydration of the phase [32]. 

**Figure 2:**
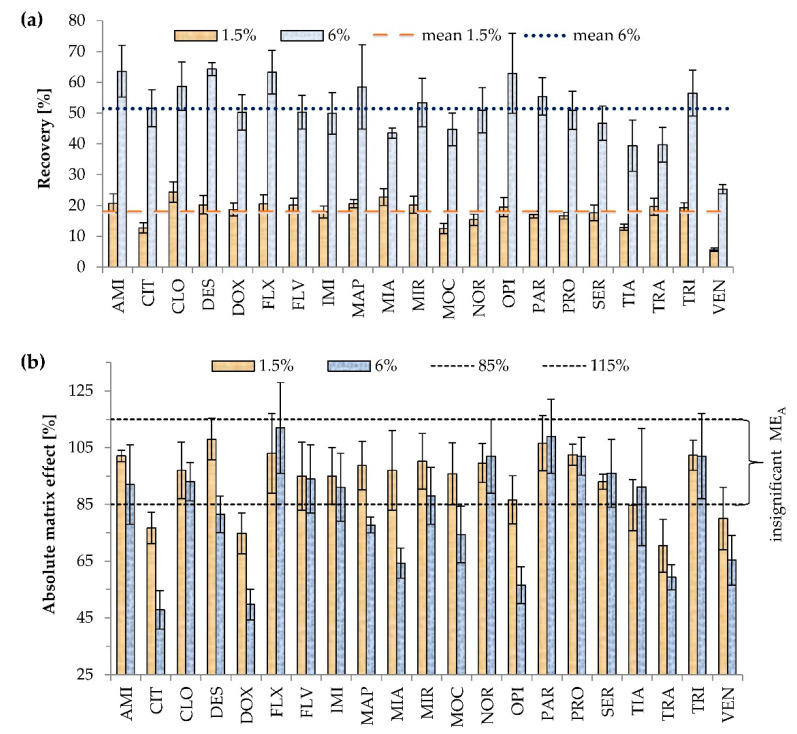
Effects of Triton X-114 concentration on (**a**) recovery (*p* < 0.001), (**b**) absolute matrix effect (ME_A_) (*p* = 0.182). Extraction conditions: equilibrium temperature—60°C; equilibrium time—20 min; sample pH 6.8; ammonium acetate content—20% (*w/v*). Antidepressants level corresponds to a plasma concentration of 100 ng/mL Higher mean recovery was observed with higher Triton X-114 concentration (the experiment was repeated three times) (*p* < 0.001). Results are presented as means and standard deviations.

**Figure 3:**
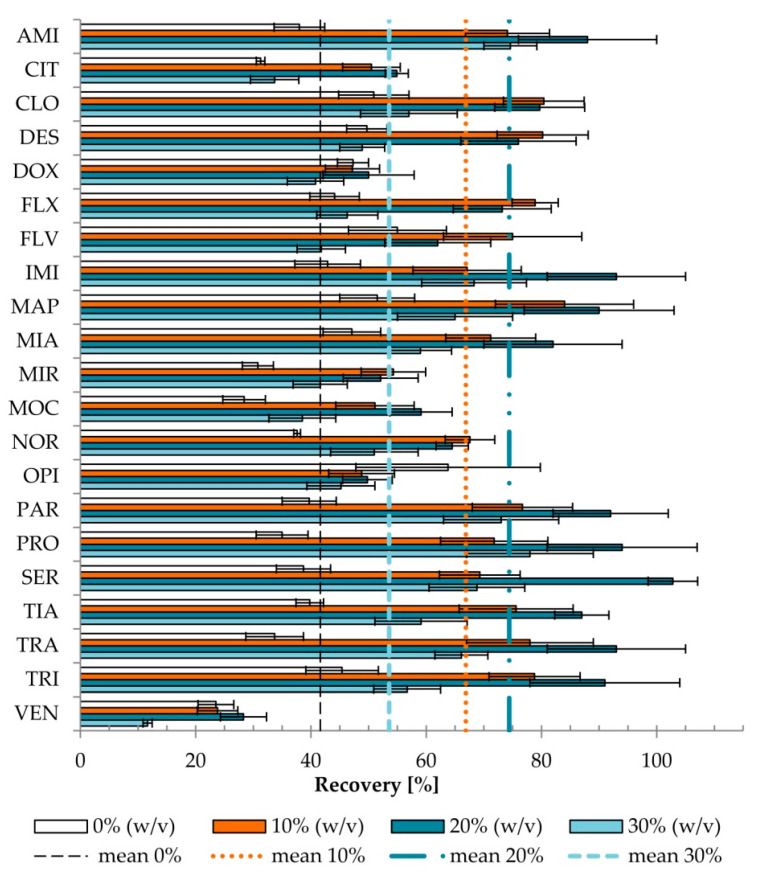
The effect of ammonium acetate concentration (0, 10, 20, and 30% (*w/v*)) on recovery (*p* < 0.001). Extraction conditions: equilibrium temperature—60 °C; equilibrium time—20 min; sample pH, 6.8; concentration of Triton X-114–6% (*w/v*). Antidepressants level corresponds to a plasma concentration of 100 ng/mL. Results are presented as means and standard deviations. Mean recoveries for 0% AA (dashed line), 10% AA (dotted line), 20% AA (dotted-dashed line), and 30% AA (bold dashed) are shown.The highest increases of recovery, as a result of AA addition, were observed for AMI, PAR, PRO, TIA, and TRA, whereas the weakest effect was recorded for both FLV and MIA. No increase in recovery for VEN or decrease for OPI was observed. The increase of recovery (10% *vs*. 0 % (*w/v*)) was correlated (r_s_ = 0.465, *p* = 0.034) with the calculated octanol–water partition coefficient (cLogP).

**Figure 4:**
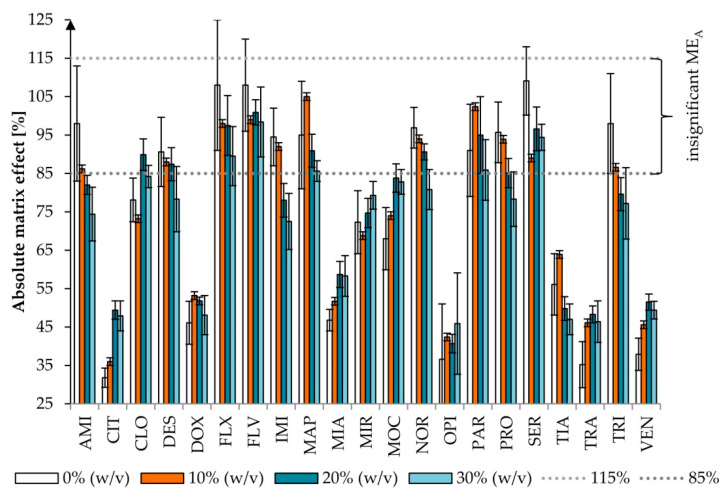
Effect of the addition of ammonium acetate on the absolute matrix effect (ME_A_) (*p* = 0.198). Extraction conditions: equilibrium temperature—60°C; equilibrium time—20 min; sample pH—6.8; concentration of Triton X-114—6% (*w/v*). Antidepressants level corresponds to a plasma concentration of 100 ng/mL. Results are presented as means and standard deviations. ME_A_ between 85% (dotted line) and 115% (dotted line) was considered insignificant.

**Figure 5:**
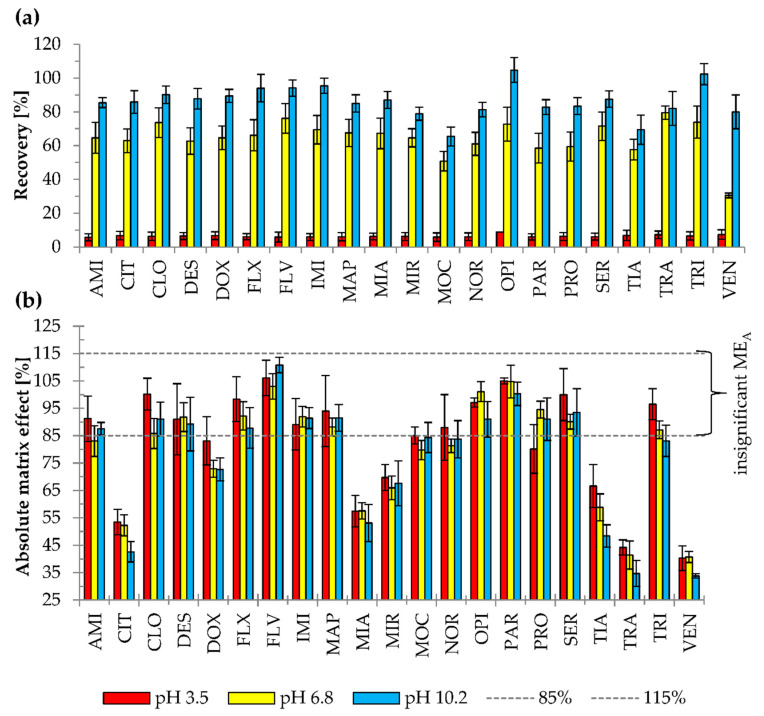
The effects of different pH of a sample on (**a**) recovery (*p* < 0.001), (**b**) absolute matrix effect (ME_A_) (*p* = 0.514). Results are presented as means and standard deviations. ME_A_ between 85% (dashed line) and 115% (dashed line) was considered as insignificant. Extraction conditions: Triton X-114 at 6% (*w/v*); ammonium acetate at 10% (*w/v*); equilibrium temperature—60°C; equilibrium time—20 min. Antidepressants level corresponds to plasma concentration of 100 ng/mL.

**Figure 6:**
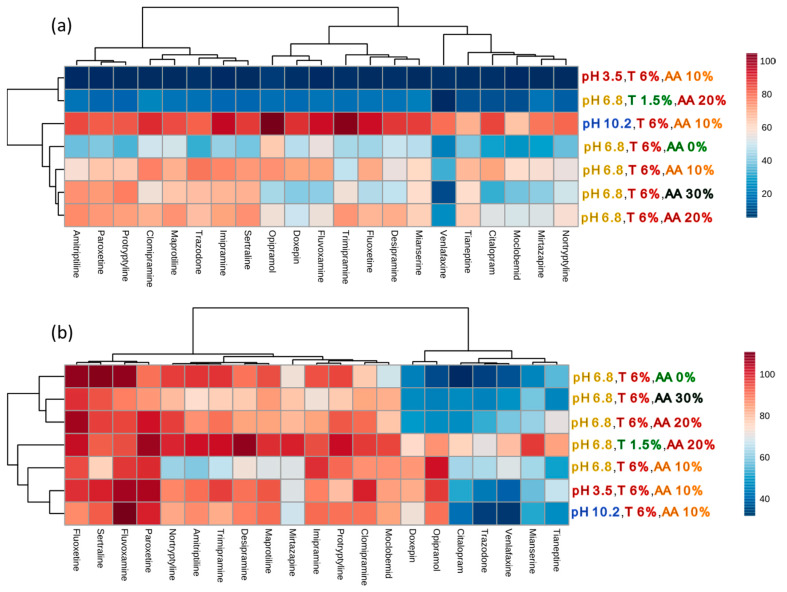
The effects of cloud-point extraction conditions on (**a**) the recoveries and (**b**) the absolute matrix effects of 21 antidepressants determined using liquid chromatography coupled with mass spectrometry. T—Triton concentration (*w/v*), AA—ammonium acetate concentration (*w/v*).

The optimal conditions (concentration of Triton-X-114 and AA, pH sample) were defined as the ones showing the highest mean recovery and the lowest absolute ME (ME_A_) (Equation (1)) [33], for simultaneously analyzing all 21 antidepressants. The influences of selected parameters are presented in Figure 2, Figure 3, Figure 4 and Figure 5 and summarized in Figure 6. The chromatography separation was not optimized. We used the gradient elution mode, eluent flow rate, and analytical column commonly used in our analysis [31]. 

#### 2.1.1. The Effects of Triton X-114

The concentration of Triton X-114, used as the surfactant in CPE, was very carefully selected. This was because the overly low concentration of Triton X-114 decreases recovery and prevents effective separation of the aqueous and micellar phases. On the other hand, too high concentration of surfactant is associated with a high volume of the micelle-rich phase, and the unwanted dilution of the analyte [5,10,32]. The range of tested Triton X-114 concentrations, added to samples in multi-compound determination, varied from 1.5 to 9% (*w/v*) [1,2,4,5,8,32], with the most frequently used concentration being 4% [32]. In this study, two variants of Triton concentration (1.5 and 6%) were studied. The effects of the surfactant concentration on recovery and ME_A_ are shown in Figure 2.

The volume of the micellar rich phase was about 100 μL, and the initial volume of the sample was 1 mL, so the analytes were concentrated about 10 times. Over twofold increases in recovery at higher Triton X-114 concentration were noted for CLO, DOX, FLV, IMI, MAP, MIR, SER, and TRA; over threefold increases for AMI, CIT, DES, FLX, MOC, NOR, OPI, PAR, PRO, and TIA; and the highest for VEN (4.5 times). A lower than twofold increase was noted only for MIA. Higher recoveries at 6% Triton were expected based on the partition coefficient equation. The partition coefficient is characteristic for the compound and the extraction conditions (e.g., type of surfactant, pH). It is calculated as the ratio of antidepressant concentration in the surfactant to that in the aqueous phase [34]. Thus, with the increase of the surfactant rich phase volume, the mass of the extracted compound increases as well.

Significant ME_A_ values were observed for four (CIT, DOX, TRA, and VEN) and nine antidepressants (CIT, DES, DOX, MAP, MIA, MOC, OPI, TRA, and VEN) out of 21 analyzed at 1.5 and 6% Triton X-114, respectively. In contrast to 6%, at 1.5% Triton X-114 concentration, ME_A_ was not observed for DES, MAP, MIA, MOC, and OPI. Higher surfactant concentration is related to a higher risk of ME_A_, as reported previously [31]. The possible reason is the interference of surfactant with droplet evaporation or higher recovery not only of analytes, but also impurities of samples that interfere with the analyte ionization. However, in the current study, the differences in the occurrence of ME_A_ for all 21 antidepressants between the two surfactant concentrations were not statistically significant (*p* = 0.182). The concentration of 6% (*w*/*v*) Triton X-114 was selected as optimal considering higher mean recovery for 21 antidepressants and comparable ME_A_. 

#### 2.1.2. The Effects of Salt Addition

Salts affect the extraction efficiency by decreasing or increasing the analyte concentration in the aqueous phase and CMC. Effects of salt addition depend on the concentrations of salts and the type of surfactant used. For the nonionic surfactants, such as Triton X-114, the value of CMC decreases with an increasing concentration of the electrolyte [35,36,37,38,39,40]. The anions (i.e., Cl^-^ and SO_4_^2-^) are likely to decrease self-association of water molecules, whereas the cation (i.e., Na^+^) may decrease the cloud point by dehydration of the polyoxyethylene chain [39,41]. Electrolytes support demulsification, which is needed to limit the amount of impurities in the final surfactant-rich phase [40]. The other important effects of electrolytes are the decrease in the cloud point temperature and promotion of phase separation by increasing the density of aqueous phase [8,41]. However, overly large amounts of salts can cause the formation of three phases, as observed in our study [8].

The most frequently used concentration of different salts is 4–6% [32], increasing the recovery of the analyte by 10–20%. The opposite effect was observed if the salt concentration ranged from 7 to 10%. Improvement of extraction efficiency associated with salt addition was probably due to the salting-out effect, which reduces the amount of water to dissolve the analyte. While the overly high concentration of salt will competitively carry substances into the protein deposition, it can lower the concentration of drug in the solution and will have an impact on the recovery [41]. Even if the addition of salt increases the recovery, non-volatile salts contaminate the ion source in LC–MS. Thus, we tested the addition of the volatile salt (AA) in three variants: 10, 20, and 30% (*w*/*v*) (sample pH 6.8, 6% (*w*/*v*) of Triton X-114). The influences of various concentrations of salt added on the recovery and ME_A_ were evaluated (Figure 3 and Figure 4)**.**

There was a statistically significant difference (*p* < 0.001) in recovery depending on the concentration of AA used in the tested conditions (sample pH 6.8, 6% Triton X-114). The differences occurred between all sample variants. The addition of 10% (*w*/*v*) AA increased recovery 1.64 times on average (range 0.87–2.31), while 20% (*w*/*v*) AA increased recovery 1.85 times on average (range 0.88–2.76), compared with the sample without the addition of AA.

The addition of 30% (*w/v*) AA resulted in lower recovery than the addition of 10% or 20% (*w/v*) of AA. Moreover, for 20 and 30% (*w/v*) of AA, three phases were observed (from the bottom of the tube: an aqueous phase, a micelle-rich phase, an aqueous phase), unlike for 0 and 10% (*w/v*) of AA, for which only two phases were formed (lower layer—micelle-rich phase; and aqueous surfactant—lean phase above). To separate the micelle-rich phase, an upper surfactant-lean phase was decanted. In the cases of 20 and 30% (*w/v*) of AA, the microsyringe should be used to remove the lower aqueous surfactant-lean phase from the bottom of the test tube. Thus, although the mean recovery was higher for 20% (*w/v*) AA, the addition of 10% (*w/v*) AA was selected as the most convenient concentration. Similar observations of the influence of electrolytes on the location of the micellar phases in the tube were reported [35]. 

ME_A_ can be observed as signal suppression (below 100%) or an enhancement (above 100%) in the presence of a matrix. Significant ME_A_ <85% or >115% was observed for 10, 10, 12, and 16 out of 21 studied drugs for 0, 10, 20, and 30% (*w/v*) of AA, respectively. The differences were not statistically significant (χ^2^ test, *p* = 0.198), nor were they in statistical analysis performed only for the compounds with significant ME_A_ (*p* = 0.244). In all variants of AA, insignificant ME_A_ was observed for five compounds: FLX, FLV, MAP, PAR, and SER. For 0 and 10% (*w/v*) of AA, that was additionally observed for AMI, IMI, and TRI, whereas for 0, 10, and 20% (*w/v*) of AA, that was the case for DES, NOR, and PRO. 

The effect of salt adding (sodium chloride) on the recovery of only one antidepressant venlafaxine in the CPE procedure was reported for the concentration range 0.1–0.5 M. The concentration of NaCl 0.3 M was chosen as optimal [8]. In this work, AA was used first time in CPE procedure combined with LC–MS not only for venlafaxine determination but also other 20 antidepressants. As the optimal concentration of salt, 1.3 M AA (10% (*w/v*) of AA) was selected.

#### 2.1.3. The Effects of pH

Depending on the sample pH, analytes can be either charged or uncharged. That also depends on the chemical properties of said analytes. The interaction of the analyte with the micellar aggregate formed by a nonionic surfactant is weaker for ionic than the neutral form. Thus, the highest recovery can be observed at pH where all analytes are uncharged and partitioned into the rich-micellar phase of Triton X-114 or another nonionic surfactant [37,42].

The effect of sample pH was examined using 6% (*w/v*) Triton X-114 and 10% (*w/v*) AA at pH 3.5, 6.8, and 10.2 (Figure 5). The mean recoveries were 6.4, 64.7, and 86.2% for acidic, neutral, and alkaline conditions, respectively. The majority of antidepressants studied have basic and lipophilic properties. In alkaline pH, more particles occur in molecular form; thus, higher affinity to nonionic micelles and higher extraction recovery were observed. At acidic pH, the majority of the antidepressants were in ionic form and had their lowest affinity for nonionic micelles. That resulted in the loss of some of the analytes during phase separation and low extraction efficiency. Moreover, the separation of two phases—the aqueous and micelle-rich ones—in the acidic and neutral pHs, was not as pronounced as in alkaline pH.

Statistical analysis (*p* < 0.001) indicated significant differences in recovery of the extraction performed at various pH values. However, no relationship between the pH of the extraction environment and the number of compounds exhibiting significant ME_A_ was observed (test χ^2^; *p* = 0.514). Statistical analysis carried out for compounds with significant ME_A_ (ANOVA, *p* = 0.013) showed statistically significant differences in ME_A_ between pHs. The results of the post hoc test revealed that these differences occurred between the alkaline and acidic environment (*p* = 0.004). The mean ME_A_ was 6.1% lower for samples with a pH of 3.5 than those of pH 10.2. However, it should be emphasized that in acidic conditions, the basic compounds have less affinity to nonionic surfactant micelles, which resulted in the lower analytical signal and lower recovery. Moreover, the separation into two phases was not distinct in acidic pH or neutral pH, which caused the loss of analytes during phases separation. Thus, pH 10.2 was selected as optimal to use in the final method.

Madej and Persona [2] developed a CPE to extract basic compounds (pKa 9.0−9.5) at pH 12, with Triton X-114 at 3.25% (*w/v*), without salt addition. The recoveries were 26.2, 81.4, 77.3, and 98.5% for paracetamol, amitriptyline, clomipramine, and promazine, respectively [2]. In this paper, the number of isolated compounds from plasma in the basic environment (pH 10.2) was much higher (*n* = 21 *vs. n* = 4) and the pKa range was wider (pKa 6.0−10.5). A higher mean recovery (86.2%) was observed than in the reported experiment (70.9%). Compared to the recovery of particular compounds, higher recoveries were achieved for AMI (81.4 *vs.* 85.4%) and CLO (77.3% *vs.* 90.1%). OPI was extracted as a neutral compound at pH 6, which resulted in 21.8% recovery, while in this study at pH 10.2, the recovery was 102.5% [2]. Thus, the extraction at pH 10.2 and the addition of AA allow for efficient isolation of antidepressants from biological matrices by the CPE method. Additionally, the extraction environment does not significantly affect the number of compounds exhibiting a matrix effect.

#### 2.1.4. Principal Component Analysis (PCA)

**Figure 7:**
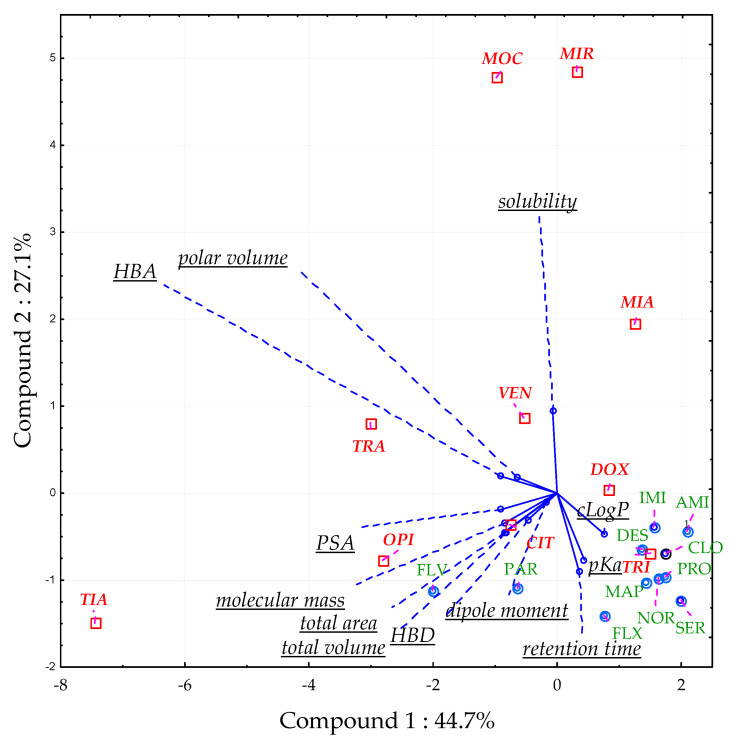
Distribution of the analyzed compounds on a score plot (principal component 2 *vs.* component 1) in a principal component analysis (PCA). The results are presented for the optimal CPE variant (pH 10.2; 6% Triton X-114, 10% (*w/v*) ammonium acetate; equilibrium temperature 60°C; equilibrium time 20 min). HBA—number of hydrogen bond acceptors, HBD—number of hydrogen bond donors, PSA—polar surface area. Compounds labelled with green text exhibited insignificant ME_A_, whereas compounds labeled with red—significant ME_A._

Some compounds revealed significant ME_A_ in all tested conditions (CIT, DOX, TRA, VEN). All of them showed a significant surfactant effect (the ratio of peak area of the analyte in a solvent with the surfactant to that without the surfactant) previously [31]. That means that the ME_A_ observed is not related to impurities extracted from plasma, but the presence of Triton in the sample. PCA was used to determine which molecular descriptors (polar volume, pKa, cLogP, dipole moment, number of hydrogen bond acceptors (HBA), number of hydrogen bond donors (HBD), total volume, molecular mass, total area, polar surface area (PSA)) influenced the ME_A_ in optimal conditions [31]. Retention time and predicted water solubility were also added to the analysis. Three principal components (PC) accounted for 82.4% of the total variation. PC1 (44.7% of the total variation) was correlated mainly with the number of HBA (−0.91), PSA (−0.90), total area (−0.85), molecular mass (−0.85), and total volume (−0.83). In turn, PC2 (27.1% of the total variation) was correlated with the retention time (−0.90), pKa (−0.77) and solubility (0.94), and PC3 (10.6% of the total variation) with the dipole moment (0.78) and HBD (0.58). The best predictors of ME_A_ occurrence were PC1 (PSA, total area, total volume, molecular mass, and the number of HBA) and PC2 (retention time, pKa, and predicted solubility) (Figure 7). 

Low polar volume; few HBA; low solubility; and high values of retention time, pKa, and cLogP indicated that the ME_A_ should not be expected. Compounds with lower lipophilicity (low cLogP and high polar volume) and low pKa were prone to significant ME_A_ [31].

### 2.2. Analytical Performance of the Proposed Method

After development, the analytical method’s performance was evaluated in terms of linearity, accuracy, precision, the lower limit of quantification (LLOQ = 10 ng/mL), and stability (autosampler, short-term, freeze and thaw). The interference from metabolites was not studied, since it occurs rarely in mass spectrometry. Due to different masses, metabolites were differentiated from parent compounds using LC–MS (Table A1). In some cases, European Medicines Agency (EMA) guidelines recommend the evaluation of the possibility of back-conversion of a metabolite into the parent analyte during the successive steps of the analysis. However, it is performed only when relevant (i.e., in case of potentially unstable metabolites—e.g., acidic metabolites to esters, unstable N-oxides, or glucuronide metabolites, lactone-ring structures) [43]. It is not an issue in the case of antidepressants.

#### 2.2.1. Linearity 

Calibration standards of eight concentrations of all analytes were prepared in blank plasma. The curves (*n* = 6) were obtained by weighted linear regression analysis with *w* = 1/x. The regression parameters for all analytes were described by the equation: y = ax + b. The values of a, b, and r^2^ for all analytes are presented in Table A2. Good linearities covered the range of 10–750 ng/mL with a coefficient of determination (r^2^) > 0.990 were obtained for all analytes regarding the peak area ratio of every analyte to internal standard (IS)versus the nominal concentration.

The linearity range of the method corresponds to therapeutic concentrations of determined antidepressants in human plasma and is similar to the reported ranges [20,21,22,28,29].

#### 2.2.2. Limit of Quantification, Precision, and Accuracy

**Figure A1:**
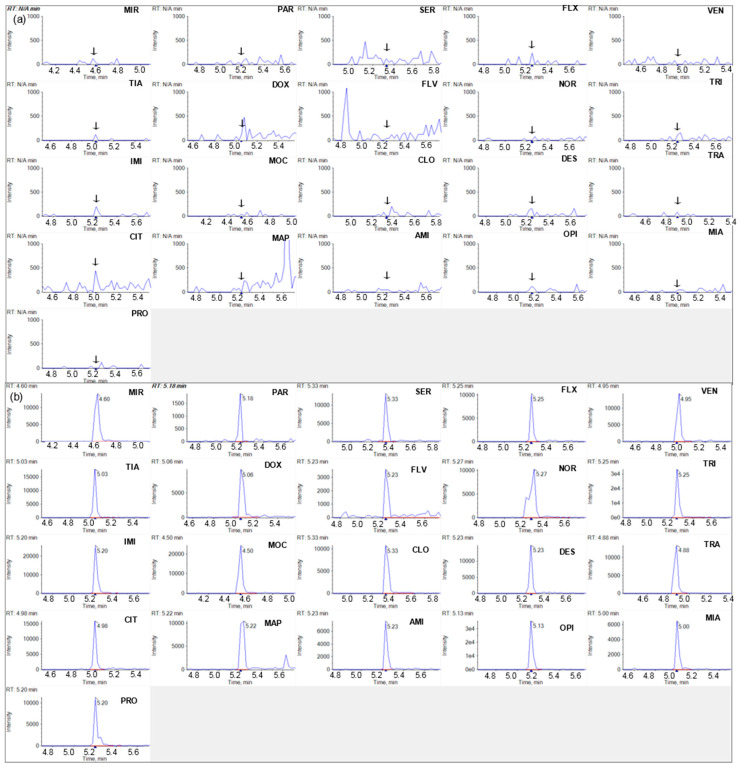
Multiple reaction monitoring (MRM) chromatogram peaks of the analyzed antidepressants extracted from (**a**) blank plasma; (**b**) quality control at a concentration of 625 ng/mL using cloud-point extraction. The MS parameters of MRM mode are presented in Table 3.

Intra-run and between-run accuracy and precision of the method for LLOQ (10 ng/mL) and QC (quality control; 16, 375, and 625 ng/mL) samples for all analytes were determined. For each LLOQ, a signal-to-noise ratio (S/N) higher than five (from 7.0 to 76.1) was observed. The accuracy was within the acceptance criteria of 85–115 and 80–120% for each QC sample and LLOQ, respectively. The precision was within the acceptance criteria, < 15 and < 20% for QC and LLOQ respectively. Precision was in the range 0.6–14.0%, and accuracy was from 85 to 114% (Table 1). Chromatograms of the analyzed antidepressants extracted from blank plasma and high QC are presented in Figure A1.

The results were repeatable and reproducible and met the criteria of the EMA [43] and the U.S. Food and Drug Administration [44] in the guidelines on bioanalytical method validation. The obtained precision is comparable to the reported values for the other extraction methods of isolating the antidepressants from plasma [20,21].

#### 2.2.3. Stability

All analytes and IS were stable under all tested conditions: autosampler stability (stability in the extract, 48 h at 4°C); and stability in human plasma, i.e., short-term stability (3 h at room temperature), and freeze-thaw stability (3 cycles at −20 °C). The QC (16 and 625 ng/mL) samples showed no significant changes in comparison to nominal concentrations. In all cases, accuracy and precision were found to be within the acceptable limits of ± 15% in both cases, as shown in Table A3.

#### 2.2.4. Matrix Effect and Recovery

The significant matrix effect was observed for eight and seven out of 21 antidepressants, for 16 and 625 ng/mL, respectively. However, since the effect was compensated by the addition of the internal standard, it did not affect the method’s accuracy and precision. It was proven by the variability of relative matrix factor (MF). The calculated coefficient of variation (CV) of the IS-normalized MF did not exceed 15% for all tested compounds (Table A4). The mean CV values were 10.2% (5.7%–15.0%) and 7.1% (2.6%–13.9%) for the concentrations of 16 and 625 ng/mL, respectively. The maximum mean CV equal to 15.0% was obtained for MIA and VEN for low QC (Table A4). The hemolysis and lipemia did not influence the method reliability.

We compared the mean ME_A_ with the values reported using other extraction techniques. The values were 122% (96–156%) and 110% (101–125%) for LLE [20], and 93.5% (47.5–115.6%) and 88.1% (48.0–107.8%) for CPE for 17 compounds at lower and higher concentrations, respectively. For five compounds extracted using SPE, the values were 102% (88–121%) and 102% (99–108%) [22] compared to 99.6% (58.2−115.6%) and 92% (54.2–107.8%) for CPE at lower and higher concentrations, respectively. The mean matrix effect of 89% (82-94%) for 12 compounds extracted using on-line SPE (1000 ng/mL) [21] was observed, whereas for CPE (625 ng/mL) was 93.2% (44.5–107.8%). Thus, CPE has a comparable mean ME_A_ to the other extraction methods, such as LLE or SPE. However, some compounds revealed significant ion suppression. 

**Figure A2:**
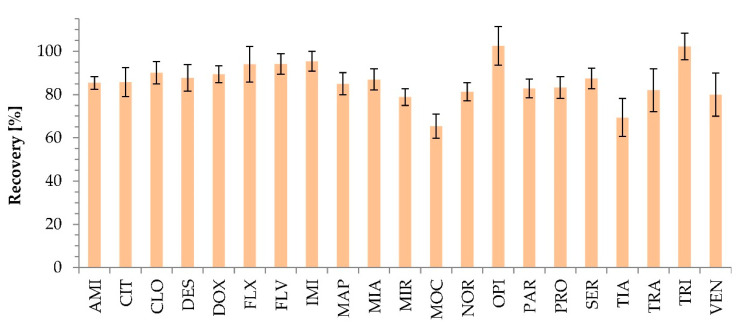
Recovery in the selected CPE method conditions (pH 10.2, 6% Triton X-114, 10% (*w*/*v*) ammonium acetate) determined for 6 different sources of plasma.

The efficiency of the extraction is determined by recovery. Recovery higher than 80% was noted for 85% of the test compounds (Figure A2). Thirteen out of 21 compounds, AMI, CIT, CLO, DES, DOX, FLX, FLV, IMI, MAP, MIA, OPI, SER, and TRI, showed satisfactory (85 to 115%) recovery. The lowest extraction efficiency was obtained jointly for MOC (65.4%) and TIA (69.4%). 

In comparison to the classic LLE developed by Fernández et al. (2012) including AMI, CIT, CLO, DES, DOX, FLX, FLV, IMI, MAP, MIA, MIR, MOC, NOR, PAR, SER, TRA, and VEN [20], mean recovery obtained for the CPE method in our study was 10.6% higher. The SPE extraction for SSRIs such as CIT, FLX, FLV, PAR, and SER provided recovery within 71–85% (mean 80%) [22], while the developed CPE extraction of these compounds was 83–94% (mean 89%). In another modification of the SPE method for AMI, CIT, CLO, DES, FLX, FLV, IMI, NOR, PAR, TRA, and VEN, the recovery was 99.6–99.9% (mean 99.8%) [21]. The CPE method developed guarantees the recovery of these compounds at the level of 80.0–95.4%, on average 87.2% (Table 2).

## 3. Materials and Methods 

### 3.1. Materials

Four lots of human plasma with EDTA as an anticoagulant were obtained from the Regional Blood Donation and Blood Therapy Centre (Warsaw, Poland). Besides, plasma with visible hemolysis and plasma with visible lipemia were obtained from the Public Central Teaching Hospital (Warsaw, Poland). 

### 3.2. Reference Standards and Chemicals

Reference standards (*n* = 21, purity ≥ 98%) (AMI, CIT, CLO, DES, DOX, FLX, FLV, IMI, MAP, MIA, MIR, MOC, NOR, OPI, PAR, PRO, SER, TIA, TRA, TRI, and VEN) were purchased from Sigma-Aldrich (St. Louis, MO, US = Merck KGaA, Darmstadt, Germany). The isotope-labeled standard (purity ≥ 98%) FLX-d5 and SER-d3 were purchased from Toronto Research Chemicals, VEN-d6 (0.1 mg/mL in methanol) from LoGiCal (LGC, Luckenwalde, Germany). Ammonia solution 25% and ethyl alcohol 96% were obtained from Avantor Performance Materials (Gliwice, Poland). Methanol and acetic acid glacial (anhydrous for analysis, EMSURE^®^) were purchased from Merck KGaA (Darmstadt, Germany). Ammonium acetate was obtained from Chempur (Piekary Śląskie, Poland). Surfactant Triton^TM^X-114 was obtained from Sigma-Aldrich Co (Merck KGaA, Darmstadt, Germany).

### 3.3. Chromatographic and Mass Spectrometric Conditions

Instrumental analysis was performed on an Agilent 1260 Infinity (Agilent Technologies, Santa Clara, CA, US), equipped with an autosampler, degasser, and binary pump coupled to a hybrid triple quadrupole/linear ion trap mass spectrometer QTRAP 4000 (ABSciex, Framingham, MA, US). The Turbo Ion Spray source was operated in the positive mode. The ion spray voltage and source temperatures were 5000 V and 600 °C, respectively. The curtain gas, ion source gas 1, ion source gas 2, and collision gas were set at 345 kPa, 207 kPa, 276 kPa, and ”high” instrument units, respectively [4]. The target compounds were optimized and analyzed in the Multiple Reaction Monitoring (MRM) mode (Table 3). 

Chromatographic separation was achieved with a Kinetex C18 column (100 mm × 4.6 mm, 5μm, Phenomenex, Milford, US) and gradient elution: (%B) 0 min 20%, 1 min 20%, 3 min 95%, 9 min 95%. [4,31]. Phase A consisted of HPLC grade water with 0.1% (*v/v*) formic acid, whereas phase B was methanol and 0.1% (*v/v*) formic acid. The flow rate was 0.75 mL/min. Total time of chromatographic analysis was 11 min, including 2 min for re-equilibration. The injection volume was 10 μL.

### 3.4. Preparation of Solutions

The surfactant Triton X-114 solutions were prepared at 5 and 20% (*w/v*) and were used to obtain final surfactant concentrations of 1.5 and 6%, respectively. The standard stock solutions of antidepressants were made by dissolving 10 mg of the reference standard in 10 mL of methanol and were stored at −39 °C. The standard working solutions of 25 μg/mL for the validation and 500 ng/mL for development were prepared by mixing equal volumes of 21 different stock solutions and the appropriate volumes of water. 

The internal standard stock solutions SER-d3, FLX-d5 (1 mg/mL), and VEN-d6 (0.1 mg/mL) were prepared in methanol. Each internal standard working solution (500 ng/mL) was prepared by mixing the internal standard stock solution with an appropriate volume of water.

FLX-d5 was an internal standard for AMI, DES, DOX, FLX, FLV, IMI, MAP, MIR, OPI, PAR, PRO, and TRI; SER-d3 was an internal standard for CLO, MIA, MOC, NOR, and SER, and VEN-d6 was an internal standard for CIT, TIA, TRA, and VEN.

### 3.5. Sample Preparation

**Figure A3:**
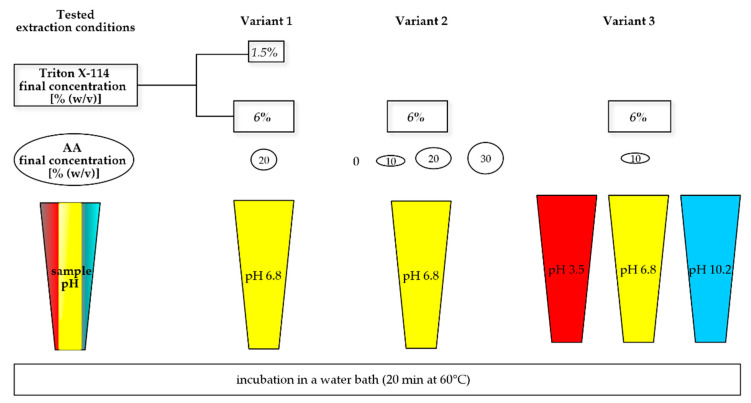
Variants of samples tested during CPE development.

The experiment consisted of the method development and validation part. All the experiments were performed in human plasma with EDTA as an anticoagulant. Tested parameters are shown in Table 4 and Figure A3. The method of extraction was the method of Kojro (2019) [31] with our modifications of Triton X-114 concentration (step a), the addition of AA (step b), and pH (step c), which was selected during the development part. 

#### 3.5.1. CPE Procedure—Development

The development was carried out in one source of plasma in 3 kinds of sample fortified by antidepressant standard-mix (50 µL of 500 ng/mL aqueous solution without the isotope-labeled standard) for all 21 analytes at a concentration corresponding to 100 ng/mL in plasma in 5 repetitions: sample A (fortified lower phase after diluting with ethanol), sample B (fortified neat ethanol), and sample C (fortified plasma before extraction). Samples A and B were used to calculate the absolute matrix effect ME_A_ [33], also called matrix factor [43] (Equation (1) in Section 3.6). The final concentrations of analytes in sample A and sample B were all the same. Samples A and C were used to assess recovery (Equation (3) in Section 3.6). 

In the first step of development, Triton X-114 at concentrations 1.5 and 6% (*w*/*v*) was tested. In the second step, the concentrations of extraction salt AA—0, 10, 20, and 30 % (*w*/*v*)—were examined; and in the third step—pHs of 3.5, 6.8, and 10.2 were tested. At every step of development, samples A, B, and C were prepared with various modifications of the studied parameters, as described in Table 4 and Figure A3.

Sample A: An aliquot of 250 μL of plasma was placed in a 1.5 mL Eppendorf tube. Then, 50 μL of water was added. The sample was vortexed for 30 s. Afterwards, 300 μL of the surfactant solution, Triton X-114 (5% or 20% to obtain the final concentration of 1.5 or 6%); 400 μL of 100% acetic acid, water, or 25% ammonia solution; and 0, 100, 200, or 300 mg AA (to obtain concentration of 0, 10, 20 or 30% (*w/v*)) were added. The sample was vortexed at low speed for 5 min and incubated in a water bath for 20 min at 60 °C. After that, the phase separation was accelerated by centrifugation (10,000 g, 5 min, 25 °C). The surfactant-lean aqueous phase was decanted to obtain the surfactant-rich phase, which was diluted with an aliquot of 500 μL of ethanol and incubated for 5 min at –80 °C. Then, the sample was centrifuged (10,000 g, 5 min, 25 °C); 200 μL of supernatant was diluted with 650 µL water and mixed together with 50 µL 500 ng/mL aqueous solution mix of the antidepressants standard without internal standards.

Sample B: 200 μL of 75%-ethanol, 650 μL water, and 50 μL 500 ng/mL aqueous solution of standard-mix were added. 

Sample C: Like sample A with modifications: (1) instead of 50 μL water, 50 μL of a 500 ng/mL aqueous solution of antidepressant standard-mix was added to 250 μL plasma; (2) finally, to the vial, 700 μL of water was added instead of 650 μL water and 50 μL of antidepressants standard-mix solution.

#### 3.5.2. CPE Procedure—Validation 

An aliquot of 250 μL of human plasma fortified with a solution of mixed standards of 21 antidepressants in appropriate concentrations from QC samples and calibration samples was placed in a 1.5 mL Eppendorf tube. Then, 50 μL of 500 ng/mL aqueous internal standard solution was added. The sample was vortexed for 30 s. Afterward, 300 μL of the surfactant 20% Triton X-114, 400 μL of the 25% ammonia solution, and 100 mg AA (to final concentration of 10% (*w*/*v*)) were added. The sample was vortexed at low speed for 5 min and incubated in a water bath for 20 min at 60 °C. The phases were centrifuged and separated (10,000 g, 5 min, 25 °C); the surfactant-lean phase was decanted. The surfactant-rich phase was mixed with a 500 μL aliquot of ethanol and incubated for 5 min at −80 °C. Then, the sample was centrifuged (10,000 g, 5 min, 25 °C). An aliquot of 200 μL of supernatant was diluted with 700 µL of water and analyzed.

#### 3.5.3. Preparation of Calibration Standards and Quality Samples for Validation Optimal CPE Conditions

The calibration standards contained all analytes at eight concentrations ranging from 10 to 750 ng/mL. The quality control samples were prepared at concentrations of 16, 375, and 625 ng/mL. All calibration standards and the quality control samples were prepared via the appropriate dilutions of blank human plasma spiked with all analytes in working solutions and were stored at ≤−39 °C. The precision was calculated as the ratio of standard deviation/mean (%). The accuracy was calculated as the determined value divided by the nominal value and expressed in percent.

Samples A and B for the matrix effect and samples A and C for the recovery test were prepared at two concentrations for all analyte standards and for IS at the working concentration in blank human plasma samples derived from six sources. 

### 3.6. Matrix Effect and Recovery Calculation

ME calculated as ME_A_ [33], also called matrix factor (MF) (Equation (1)) [43], was regarded as insignificant if included between 85 and 115%. In development, the samples for matrix effect were prepared from blank human plasma from one source at a level corresponding to a plasma concentration of 100 ng/mL, so the matrix factors were calculated as ME_A._ The preparation of samples A, B, and C was described in Section 3.5.1. In validation, the MF were calculated as the ratios of the instrument response for substances in sample A and sample B at two concentrations (16 and 625 ng/mL) for all analyte standards and for IS in the working concentration in 6 different sources, including haemolyzed and hyperlipidaemic plasma. The CV of the IS-normalized matrix factor (Equation (2)) should not exceed 15%.

Recovery was calculated by dividing the analytical signal pre-extraction spiked sample plasma (sample C) by post-extraction spiked sample plasma (sample A) (Equation (3)). Sample C concentrations were calculated on account of the volume of the rich-micellar phase.

(1) $${\mathrm{ME}}_{A}=\mathrm{MF}=\frac{\mathrm{peak}\;\mathrm{area}\;\mathrm{of}\;\mathrm{the}\;\mathrm{analyte}\;\mathrm{in}\;a\;\mathrm{matrix}\;(\mathrm{sample}\;A)\;}{\mathrm{peak}\;\mathrm{area}\;\mathrm{of}\;\mathrm{the}\;\mathrm{analyte}\;\mathrm{in}\;a\;\mathrm{solvent}\;(\mathrm{sample}\;B)}\times \;100\%$$

(2) $$\mathrm{IS}-\mathrm{normalised}\;\mathrm{MF}=\frac{\mathrm{MFanalyte}}{{\mathrm{MF}}_{\mathrm{IS}}}$$

During development, recovery was calculated according to Equation (3).

(3) $$\mathrm{Recovery}=\frac{\mathrm{peak}\;\mathrm{area}\;\mathrm{of}\;\mathrm{the}\;\mathrm{analyte}\;\mathrm{in}\;\mathrm{variant}\;\mathrm{sample}\;C}{\mathrm{peak}\;\mathrm{area}\;\mathrm{of}\;\mathrm{the}\;\mathrm{analyte}\;\mathrm{invariant}\;\mathrm{sample}\;A}\times 100\%$$

### 3.7. Statistical Analysis

The statistical analysis of the results was performed with the STATISTICA version 12 for Windows (TIBCO Software Inc., Palo Alto, CA, USA). The normal distribution of the results was evaluated by the Shapiro–Wilk test. For the normal distribution, the t-Student test and ANOVA were used.

The one-way ANOVA followed by the LSD post hoc test for multiple comparisons of the dependent group was applied. Differences between two nominal variables were tested using the chi-square test (χ^2^). A *p*-value below 0.05 was considered significant.

PCA was used to find molecular descriptors that divided the studied group into prone and not prone to the ME.

## 4. Conclusions

The use of a volatile salt (1.3 M–10% (*w/v*) of AA) is a promising modification of the CPE procedure for LC–MS applications, since the mean recovery of studied antidepressants increased almost two-fold. The procedure can be applied to other groups of analytes to develop reliable and environmentally friendly screening methods. In the future, other volatile salts such as ammonium formate can be used for comparisons. Non-volatile salts can be applied in CPE-LC/MS when the discharge of the initial part of the chromatographic course is possible. The modified CPE method is an alternative for LLE and SPE for sample preparation prior to LC–MS, giving comparable recovery and matrix effect. Using ethanol instead of methanol or acetonitrile in CPE to reduce the sample viscosity at the last point of sample preparation makes the method more friendly to the environment. The selected conditions of CPE extraction of antidepressants were as follows: 6% (*w/v*) Triton X-114, 10% (*w/v*) AA, and pH 10.2. The developed CPE–LC–MS method was proven to be precise, accurate, and reliable, and can be applied for the simultaneous determination of various antidepressants in human plasma in clinical and forensic toxicology.

Limitation of the study: The method was not applied to clinical samples. Not all parameters that can influence the method performance were tested, e.g., extraction temperature, equilibration time, types of volatile salts, and anticoagulants used in blood sample collection. 

## Figures and Tables

**Table 1:** Precision and accuracy intra-run (*n* = 5) and between-run (*n* = 15) in human plasma for lower limit of quantification (LLOQ), low QC, medium QC, and high QC.

		LLOQ 10 ng/mL		Low 16 ng/mL		Medium QC 375 ng/mL		High QC 625 ng/mL	
		Precision	Accuracy	Precision	Accuracy	Precision	Accuracy	Precision	Accuracy
Compound		[%]	[%]	[%]	[%]	[%]	[%]	[%]	[%]
Amitriptyline	intra-run	1.5	92	5.5	107	5.9	86	5.4	98
	between-run	8.2	104	6.4	103	2.3	88	1.5	99
Citalopram	intra- run	6.1	93	4.8	90	7.7	89	6.1	109
	between-run	2.6	93	3.4	90	5.3	86	10	99
Clomipramine	intra- run	6.7	88	3.7	98	10	104	9.1	105
	between-run	9	100	5.4	98	3.3	101	3	108
Desipramine	intra- run	5.1	106	5.6	101	11	92	5.6	95
	between-run	14	102	6.9	99	3	89	4.1	99
Doxepin	intra-run	3.4	104	6.8	99	7.6	110	8.4	113
	between-run	6.2	110	3	96	2.5	103	5	109
Fluoxetine	intra-run	6.7	108	5.4	109	8.1	91	0.8	97
	between-run	0.8	109	0.6	108	0.3	91	0.4	98
Fluvoxamine	intra-run	5.9	108	9.6	102	7.8	108	5.6	109
	between-run	11	85	4.3	95	1.3	100	9	112
Imipramine	intra-run	4.3	94	3.3	99	7.7	85	6.7	104
	between-run	9.3	89	13	92	4.3	89	0.3	112
Maprotiline	intra-run	2.7	96	6.1	89	12	92	7.1	104
	between-run	7.9	97	5.2	88	1.2	93	0.7	105
Mianserin	intra-run	3.8	113	3.9	101	8.8	114	11	93
	between-run	2.9	112	3.5	96	14	97	11	105
Mirtazapine	intra-run	6.2	97	13	101	11	95	5	100
	between-run	12	102	6.2	104	2.8	93	7.2	107
Moclobemide	intra-run	2.4	102	4.3	85	11	90	11	98
	between-run	11	110	7.9	89	4.4	86	1.6	96
Nortriptyline	intra-run	8.7	93	6.9	102	8.1	102	4.1	110
	between-run	9.6	95	14	99	6.7	109	0.2	110
Opipramol	intra-run	5.6	93	5.2	90	3.2	92	9.2	105
	between-run	10	101	3.6	87	3.5	89	4.7	110
Paroxetine	intra-run	7.5	105	7.4	101	12	100	11	91
	between-run	4.5	106	2.9	102	0.8	99	6.7	97
Protriptyline	intra-run	3.7	94	4.8	97	11	90	7.3	94
	between-run	9.5	104	8.5	95	2.1	88	6.8	100
Sertraline	intra-run	4.4	109	2	104	3.2	107	5.3	105
	between-run	6.1	109	2.5	101	7	100	1.5	107
Tianeptine	intra-run	2	103	3.2	93	11	93	9	111
	between-run	3.2	106	2.9	97	1.8	95	5.8	105
Trazodone	intra-run	2.4	111	4.1	88	6.1	89	5.6	105
	between-run	9	103	3.3	92	1.6	91	2.7	102
Trimipramine	intra-run	6.1	85	3.7	98	6	88	5.3	100
	between-run	11	100	3.7	101	0.2	87	1.9	102
Venlafaxine	intra-run	5.3	106	2.4	100	9.7	92	3.3	103
	between-run	3.4	102	3	104	6	87	4.8	98

**Table 2:** Comparison of the reported recoveries of antidepressants using various methods. The arrows indicate the differences in recoveries in comparison to this study.

Study	This study	del Fernández et al. 2012 [20]				Madej and Persona 2013 [2]		Qin et al. 2008 [8]		Ansermot et al. 2012 [22]		de Castro et al. 2007 [21]	
Extraction	CPE	LLE				CPE		CPE		SPE		on-Line SPE	
Analytical Method	LC/MS	LC/MS				LC/DAD		LC/FL		LC/MS		LC/MS	
QC		Low		High									
Amitryptyline	85.4	70		67	↑	70	↑	-		-		99.9	↓
Citalopram	85.8	85		81	↑	-		-		83	↑	99.9	↓
Clomipramine	90.1	64		66	↑	-		-		-		99.7	↓
Desipramine	87.7	72		74	↑	-		-		-		99.8	↓
Doxepine	89.4	70		60	↑	-		-		-			
Fluoxetine	94.0	74		72	↑	-		-		82	↑	99.9	↓
Fluvoxamine	94.1	70		71	↑	-		-		-		99.7	↓
Imipramine	95.4	76		78	↑	-		-		-		99.9	↓
Maprotyline	85.0	77		74	↑	-		-		-			
Mianserine	87.0	86		83	↑	-		-		-			
Mirtazapine	78.9	83		84	↓	-		-		-			
Moclobemide	65.4	71		73	↓	-		-		-			
Nortryptyline	81.3	71		73	↑	-		-		85	↓	99.6	↓
Opipramol	102.5	-		-		25	↑	-		-			
Paroxetine	82.8	69		73	↑	-		-		74	↑	99.8	↓
Protryptiline	83.3	-		-		-		-		-			
Sertraline	87.5	63		70	↑	-		-		80	↑	99.9	↓
Tianeptine	69.4	-		-		-		-		-			
Trazodone	82.0	87		88	↓	-		-		-		99.9	↓
Trimipramine	102.3	-		-		-		-		-			
Venlafaxine	80.0	83		81	↓	-		89	↓	-		99.8	↓
Mean	86.2	75		75		48		89		81		99.8	
Range	65.4–102.5	63–87		60–88		25–70				74–85		99.6–99.9	

**Table 3:** The optimized parameters of MRM mode of antidepressant determination in ESI+.

Drug Name	Parent Ion [*m/z*] [M+H^+^]	Daughter Ion [*m/z*]	DP [V]	CE [V]	CXP [V]
Amitriptyline	278	233	91	25	20
Citalopram	325	109	86	39	8
Clomipramine	315	86	81	29	6
Desipramine	267	72	81	31	12
Doxepin	280	107	71	33	6
Fluoxetine	310	44	56	37	6
Fluoxetine-d5	315	44	71	41	6
Fluvoxamine	319	71	71	33	4
Imipramine	281	86	76	25	6
Maprotiline	278	250	86	27	16
Mianserin	265	208	96	31	12
Mirtazapine	266	195	46	37	10
Moclobemide	269	182	76	27	10
Nortriptyline	264	233	71	23	14
Opipramol	364	171	91	31	14
Paroxetine	330	192	51	31	14
Protriptyline	264	191	96	41	14
Sertraline	306	159	51	35	12
Sertraline-d3	309	275	56	17	16
Tianeptine	437	292	61	25	8
Trazodone	372	176	81	37	10
Trimipramine	295	100	71	25	6
Venlafaxine	278	58	56	47	10
Venlafaxine-d6	284	64	66	49	10

**Table 4:** Variants of samples tested during CPE development and validation—different variants of final concentration of surfactant Triton X-114, concentration of ammonium acetate (AA), and sample pH. Samples were incubated in a water bath for 20 min at 60 °C.

Part 1. Development—Changes in Each Part			
Variant	Triton X-114 in Sample [% (*w/v*)]	Sample pH	AA [% (*w/v*)]
Concentration of surfactant (a)	(a1) 1.5 (a2) 6	6.8 ^1^	20
Concentration of salt (b)	6	6.8 ^1^	(b1) 0 (b2) 10 (b3) 20 (b4) 30
Sample pH (c)	6	(c1) 3.5 ^2^(c2) 6.8 ^1^ (c3) 10.2 ^3^	10
**Part 2. Validation method on sample C**			
Optimal method	6	10.2 ^3^	10

^1^ water; ^2^ by acetic acid 100%; ^3^ by ammonia solution 25%.

## Appendix A

**Table A1:** Comparison of MRM transitions of the analyzed antidepressants with those of their metabolites determined in other papers using LC–MS/MS.

Drug Name	Parent ion [*m/z*] [M+H^+^]	Daughter ion [*m/z*]	Metabolite	Parent ion [*m/z*] [M+H^+^]	Daughter ion [*m/z*]	Source	Biofluid
Amitriptyline	278	233	Nortriptyline	264	90	[25]	human plasma
Citalopram	325	109	Desmethylcitalopram	311	109	[45,46]	human plasma whole blood
					262		
			Citalopram *N*-oxide	341	262		human plasma whole blood
					109		
Clomipramine	315	86	Norclomipramine	301	72	[47]	human plasma
			Demetylclomipramine	302	243 271	[48]	whole blood
			Hydroxydesipramine	282	252		
Doxepin	280	107	Nordoxepin	266	107	[49]	human plasma
Fluoxetine	310	44	Norfluoxetine	296	134	[47]	human plasma
Imipramine	281	86	Desipramine	267	72	This study	human plasma
Maprotiline	278	250	*N*-desmethylmaprotiline	264	117	[46]	human whole blood
					169		
Mirtazapine	266	195	Desmethyl mirtazapine	252	195	[50]	human bile
			8-Hydroxydesmethylmirtazapine	282	211		
Nortriptyline	264	233	10- hydroxynortriptyline	280.1	262.2	[51]	human plasma
Paroxetine	330	192	4-hydroxy-3-methoxy	332	192	[52]	human plasma
Sertraline	306	158	*N*-Desmethyl sertraline	292.32	275.1	[46]	human whole blood
Tianeptine	437	292	MC5	409	292	[53]	rat plasma
					228		
Trazodone	372	176	m-Chlorophenylpiperazine	197	118	[49]	human plasma
Trimipramine	295	100	Desmethyltrimipramine	281	86	[54]	human hair
			2-Hydroxytrimipramine	311	100		
			2-Hydroxydesmethyltrimipramine	297	86		
Venlafaxine	278	58	O-Desmethyl venlafaxine	264	58	[46]	whole blood
					246		

There is no reported MRM methods for metabolites of other analyzed antidepressants.

**Table A2:** The parameters of the calibration curve for all 21 analytes (*n* = 6).

Compound	R^2^	Linear Equation y = ax + b	Standard Deviation a	Standard Deviation b
Amitriptyline	0.998	y = 0.001731x + 0.0100	0.000062	0.0016
Citalopram	0.993	y = 0.001895x + 0.0016	0.000052	0.0011
Clomipramine	0.997	y= 0.000728x + 0.002747	0.000017	0.000940
Desipramine	0.996	y = 0.002608 ± 0.0008	0.000051	0.0015
Doxepin	0.994	y= 0.001895x + 0.0119	0.000064	0.0075
Fluoxetine	0.997	y = 0.002061x+ 0.0030	0.000075	0.0030
Fluvoxamine	0.994	y = 0.000679x + 0.0079	0.000030	0.0032
Imipramine	0.997	y = 0.00395x + 0.0054	0.00012	0.0057
Maprotiline	0.995	y = 0.002716x + 0.0159	0.000050	0.0031
Mianserin	0.996	y = 0.0002657x + (-0.000142)	0.0000077	0.000265
Mirtazapine	0.997	y = 0.003092x + (-0.0013)	0.000081	0.0050
Moclobemide	0.990	y = 0.0026x + (-0.0014)	0.0011	0.0019
Nortriptyline	0.996	y = 0.001011x + 0.000020	0.000027	0.000936
Opipramol	0.997	y = 0.0260x + (-0.010)	0.0044	0.017
Paroxetine	0.996	y = 0.0001912x + 0.0050	0.0000063	0.0027
Protriptyline	0.997	y = 0.002013x + 0.0050	0.000048	0.0040
Sertraline	0.998	y = 0.000591x + 0.0035	0.000012	0.0015
Tianeptine	0.995	y = 0.002512x + 0.0026	0.000084	0.0032
Trazodone	0.996	y = 0.002249x + (-0.0026)	0.000095	0.0025
Trimipramine	0.998	y = 0.00573x + 0.0033	0.00023	0.0046
Venlafaxine	0.999	y = 0.002268x + 0.0014	0.000086	0.0032

The uncertainty (standard deviation) was rounded up to two significant figures. The mean was rounded to have the same number of decimal places as the uncertainty.

**Table A3:** Sample stability—autosampler, short-term, and freeze and thaw stability; mean accuracy [%] (n = 5) for low and high QC.

Test Name	Autosampler Stability				Short-Term Stability				Freeze and Thaw Stability			
	(48h at 4°C)				(3h at Room Temperature)				(−20°C)			
Drug Name	16	CV (%)	625 ng/mL	CV (%)	16	CV (%)	625 ng/mL	CV (%)	16	CV (%)	625 ng/mL	CV (%)
	ng/mL				ng/mL				ng/mL			
Amitriptyline	112.2	13	98.1	10	96	9	91.9	4	103.2	8	88.4	13
Citalopram	92.9	14	91.7	12	96.9	6	102.6	4	89.5	2	96.3	8
Clomipramine	102.8	1	96.3	4	101.2	4	98.3	5	102	9	97.2	4
Desipramine	108.7	6	99.1	13	92.7	6	93.1	3	101.5	5	92.3	5
Doxepin	110.3	9	90.6	11	94.1	3	91.1	2	91.3	7	86.5	12
Fluoxetine	104.9	14	98.8	9	97.7	5	94.2	1	110	7	90.9	8
Fluvoxamine	106.7	9	102.5	5	98.3	11	100	7	91.5	13	98.9	5
Imipramine	110.6	12	98.4	8	97.1	13	92.4	3	95.9	3	91.9	6
Maprotiline	104	9	106.2	6	113.3	2	98.4	7	110.5	3	96.4	7
Mianserin	106.9	13	91.1	6	95.2	3	99.4	4	91.5	2	91.3	6
Mirtazapine	107.2	10	92.4	9	92.7	11	94.6	6	99.7	13	88.3	12
Moclobemide	108	9	90.8	10	99.3	8	98.1	7	99.6	9	91.1	4
Nortriptyline	112.6	6	105.5	9	110.5	10	100.3	5	113.3	6	97.6	8
Opipramol	101.5	14	91.7	10	102.2	3	91.5	4	99.5	5	89.1	8
Paroxetine	109.3	6	94.2	6	108.5	8	101.6	9	107.1	9	91.3	13
Protriptyline	111.4	4	101.1	9	102.1	5	91.9	4	103.2	3	88	7
Sertraline	99.6	11	99.4	12	96.9	6	100.3	6	109.2	9	99.2	3
Tianeptine	96	10	90.4	12	93.5	7	102.7	6	93	6	100.8	9
Trazodone	97.2	5	96.6	12	99.7	7	104.7	5	92.1	8	100.3	8
Trimipramine	110	12	95.9	6	89.6	4	102.6	2	101.7	11	90.3	4
Venlafaxine	101.2	8	100.4	7	95.8	7	104.7	9	97.9	12	99.8	8

**Table A4:** Matrix effect values of tested compounds for low and high QC.

QC Samples	Low QC (16 ng/mL)			High QC (625 ng/mL)		
Drug Name	MF Analyte	IS-Normalized MF	CV IS-Norm. MF [%]	MF Analyte	Is-Normalized MF	CV IS-Norm. MF [%]
Amitriptyline	98.7	92.5	10.0	94.8	94.6	6.1
Citalopram	58.2	123.3	14.6	54.2	129.8	12.1
Clomipramine	108.3	106.2	9.3	95.1	99.1	2.7
Desipramine	95.6	90.2	14.6	95.4	95.5	8.9
Doxepin	79.2	73.3	13.7	73.9	73.7	6.5
Fluoxetine	106.3	99.1	9.3	96.6	96.5	10.0
Fluvoxamine	108.8	101.4	7.9	107.8	107.8	7.7
Imipramine	100.1	93.0	6.7	94.0	93.8	4.4
Maprotiline	102.1	95.7	9.4	97.7	97.5	2.6
Mianserin	64.8	63.1	15.0	58.7	61.0	8.9
Mirtazapine	72.6	68.1	14.8	70.4	70.4	3.3
Moclobemide	88.9	87.1	6.8	89.0	93.2	5.3
Nortriptyline	94.2	92.2	5.7	92.6	96.8	3.6
Opipramol	107.9	101.4	10.9	102.4	102.4	7.7
Paroxetine	115.6	111.8	6.7	107.1	107.1	7.6
Protriptyline	104.3	97.8	9.2	97.7	97.6	5.1
Sertraline	109.1	106.9	7.2	94.4	98.5	2.8
Tianeptine	73.8	142.1	6.3	64.3	153.9	13.9
Trazodone	53.2	108.0	12.4	48.0	108.1	11.9
Trimipramine	101.7	95.0	7.7	88.7	88.5	6.8
Venlafaxine	47.5	96.5	15.0	44.5	105.5	11.6
